# Case Report: Deep brain stimulation improves tremor in *FGF-14* associated spinocerebellar ataxia

**DOI:** 10.3389/fneur.2022.1048530

**Published:** 2022-12-14

**Authors:** Moritz A. Loeffler, Matthis Synofzik, Idil Cebi, Philipp Klocke, Mohammad Hormozi, Thomas Gasser, Alireza Gharabaghi, Daniel Weiss

**Affiliations:** ^1^Department for Neurodegenerative Diseases, Centre for Neurology University Hospital and University of Tübingen, Tübingen, Germany; ^2^Hertie-Institute for Clinical Brain Research, University of Tübingen, Tübingen, Germany; ^3^Institute for Neuromodulation and Neurotechnology, Department of Neurosurgery and Neurotechnology, University Hospital and University of Tübingen, Tübingen, Germany

**Keywords:** DBS, *FGF14*, spinocerebellar ataxia, case report, SCA 27, frequency

## Abstract

**Objectives:**

Spinocerebellar ataxia 27 (SCA 27) is a rare heredodegenerative disorder caused by mutations in the *fibroblast growth factor 14* (*FGF14*) and characterized by early-onset tremor and progressive ataxia later during the disease course. We investigated the effect of deep brain stimulation (DBS) of the ventralis intermedius nucleus of the thalamus (VIM) and subthalamic projections on tremor and ataxia.

**Methods:**

At baseline, we studied the effects of high-frequency VIM stimulation and low-frequency stimulation of subthalamic projections on tremor and ataxia. The patient then adopted the best individual high-frequency stimulation programme at daytime and either 30 Hz-stimulation of the subthalamic contacts or StimOFF at night during two separate 5-weeks follow-up intervals. Both patient and rater were blinded to the stimulation settings.

**Results:**

High-frequency stimulation of the VIM effectively attenuated tremor. At follow-up, intermittent 30 Hz-stimulation at night resulted in a superior tremor response compared to StimOFF at night. Ataxia was not affected.

**Discussion:**

Stimulation of the VIM and adjacent subthalamic projections effectively attenuated tremor in a patient with confirmed SCA 27. Cycling between daytime high-frequency and night-time low-frequency stimulation led to a more sustained tremor response. This suggests to study in future if low-frequency stimulation of the subthalamic projection fibers may help overcome tolerance of tremor that is observed as a long-term limitation of VIM-DBS.

## Introduction

Spinocerebellar ataxia 27 (SCA 27) is a rare cerebellar ataxia caused by mutations in the *fibroblast growth factor 14* (*FGF14)* gene characterized by postural tremor manifesting in early adulthood and slowly progressive ataxia in later decades (1).

Deep brain stimulation (DBS) of the ventralis intermedius nucleus (VIM) and subthalamic projections harboring the dentatothalamic tract (DTT) is highly effective in essential tremor (ET) (2). However, its effect in heredodegenerative ataxias associated with tremor such as spinocerebellar ataxias (SCA) or fragile X ataxia (FXTAS) remains poorly explored (Table 1). Stimulation of subthalamic projections can induce ataxia in ET (3) as a side effect caused by antidromic activation and maladaptive plasticity of the deep cerebellar nuclei (4). Experimental data from the shaker rat, a common ataxia model characterized by neurodegeneration of cerebellar Purkinje cells, suggested that high-frequency stimulation of the dentate nucleus (DN) induced ataxia, whereas low-frequency stimulation improved ataxia and even led to a superior tremor response (5). In this context, low-frequency stimulation was hypothesized to beneficially enhance cerebello-thalamo-cortical network activity involved in the manifestation of tremor and ataxia. Recent studies in ET patients suggested that effective attenuation of tremor is facilitated by stimulation along the DTT and not just in a specific anatomical region, highlighting the essential role of the DTT in tremor-associated network disorders (2, 6, 7). On this basis, we aimed to study in a patient with confirmed SCA 27, (i) if DBS of the VIM and subthalamic projections harboring the DTT is effective in treating tremor, and (ii) if a frequency modulation approach of high (180 Hz) vs. low (30 Hz) frequencies would benefit the tremor and ataxia outcomes.

**Table 1:** DBS for the treatment of tremor in heredodegenerative ataxias.

**Genetic ataxia**	**DBS target**	**Outcome**	**References**
SCA 2	VIM/ZI	Attenuation of postural tremor in 3 patients with the TRS improving from 33–26 (Oyama et al.) to 99–26 (Isobe et al.)	(11–13)
SCA 3	DN	Significant attenuation of cerebellar tremor after active stimulation vs. sham (18.0 ± 17.2 vs. 22.2 ± 19.5; *p* = 0.039) in 2 patients with SCA3 and 3 patients showing cerebellar lesions	(14, 15)
SCA 3, type IV	STN	Alleviation of parkinsonism including resting tremor in 1 patient	(16)
SCA 6	VIM	Attenuation of action tremor in 2 patients	(17)
SCA 31	VIM	Attenuation of action tremor in 1 patient	(17)
SCA unspecified	VIM/ZI	Favorable attenuation of intention tremor by stimulation of the ZI compared to the VIM in 1 patient	(18)
FXTAS	VIM/ZI	Long-term improvement of axial and intention tremor in 10 FXTAS patients reported with variable outcome concerning the tremor scores	(12, 19–25)

DBS, deep brain stimulation; SCA, spinocerebellar ataxia; FXTAS, fragile X ataxia syndrome; VIM, ventralis intermedius nucleus; ZI, zona incerta; DN, dentate nucleus; TRS, fahn-tolosa-marin-tremor-rating-scale.

## Case description

**Figure 1:**
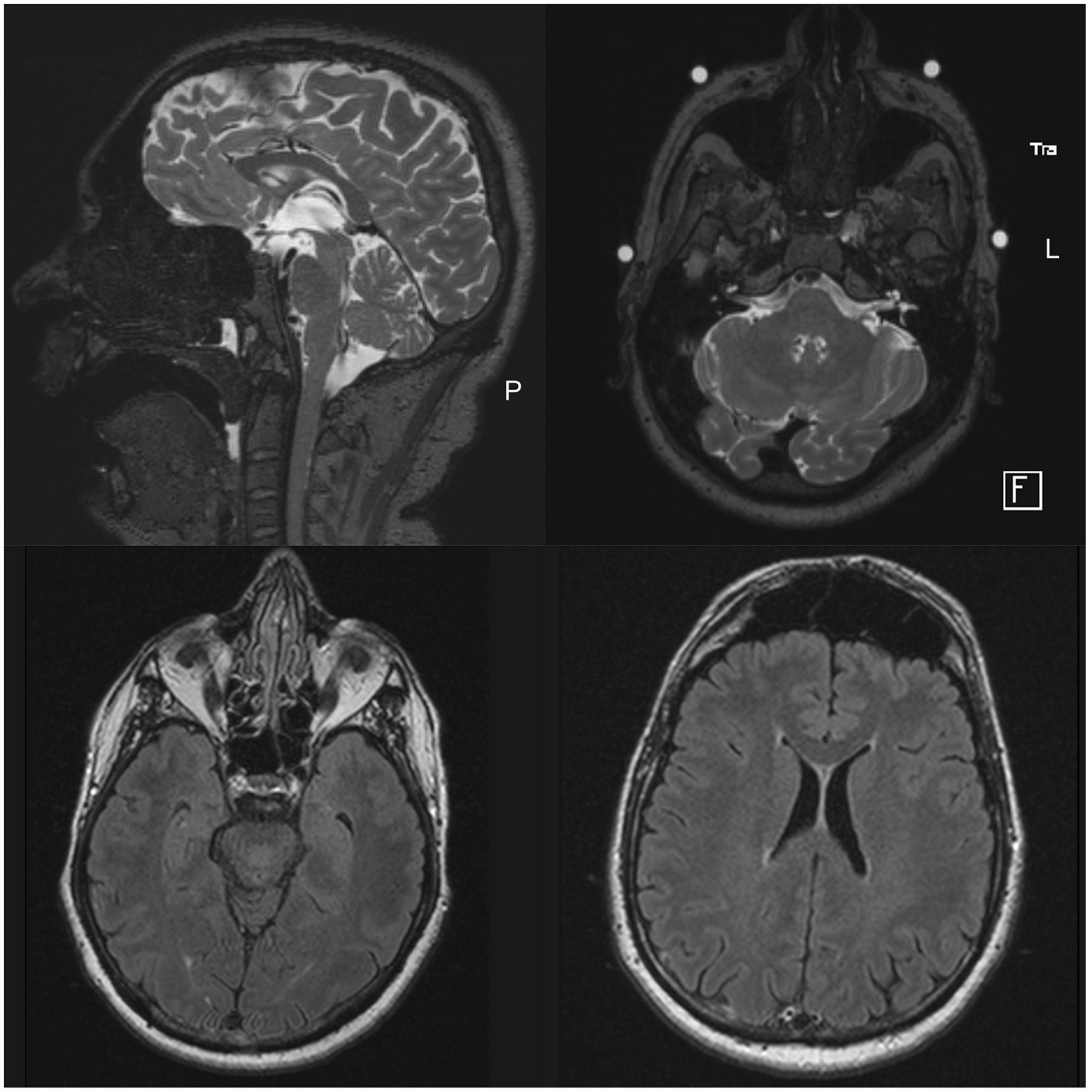
MRI scan before DBS implantation. At age 47 years, MRI showed no signs of cerebellar or supratentorial atrophy. The middle cerebellar peduncles (MCP) were normally developed according to the patient's age. The images presented in the sagittal and axial plane were acquired as T2-weighted sequences.

The male patient developed a bilateral postural arm tremor at the age of 7 years and was initially diagnosed with ET. Medication regimens including levodopa, primidone (up to 250 mg/day) and propranolol (up to 240 mg/day) did not result in relevant symptom improvement. Due to slowly progressive symptom aggravation, the patient was referred to our center for DBS implantation at the age of 47 years. He clinically presented with postural and action tremor with an amplitude of 3–5 cm including a mild intention component of the upper extremities as well as a “no-no” head tremor. No signs of gait ataxia were evident in the initial examination. Moreover, the cognitive status was assessed by a Mini-Mental State Examination (MMSE) scoring 30/30 points. Brain imaging revealed no significant supra- and infratentorial atrophy patterns. The cerebellum was developed according to the patient's age including the middle cerebellar peduncles (MCP) thereby not revealing any evidence for FXTAS (Figure 1). The family history was positive, as the patient's mother had also been diagnosed with essential tremor, having developed the same symptomatology since early childhood. In addition, she became wheelchair-bound at the age of 72 and developed dementia starting in her mid-70s, which was attributed to age-related impairments and never associated with the tremor symptomatology. The patient was implanted with quadripolar electrodes (model 3389 Medtronic). In postoperative regular reprogramming, we detected the 2nd-lowermost contact placed in the VIM to achieve best tremor control. The patient developed signs of gait and limb ataxia 2 years from surgery. Ataxia persisted after StimOFF for 96 h ruling out stimulation-induced side effects. Given this emerging persistent ataxia further diagnostic work-up was initiated with advanced copy number variant analysis of exome sequencing. A heterozygous macro-deletion of the four last exons of *FGF14* was revealed leading to the diagnosis of SCA 27.

## Timeline of the diagnostic assessment and programming

**Figure 2:**
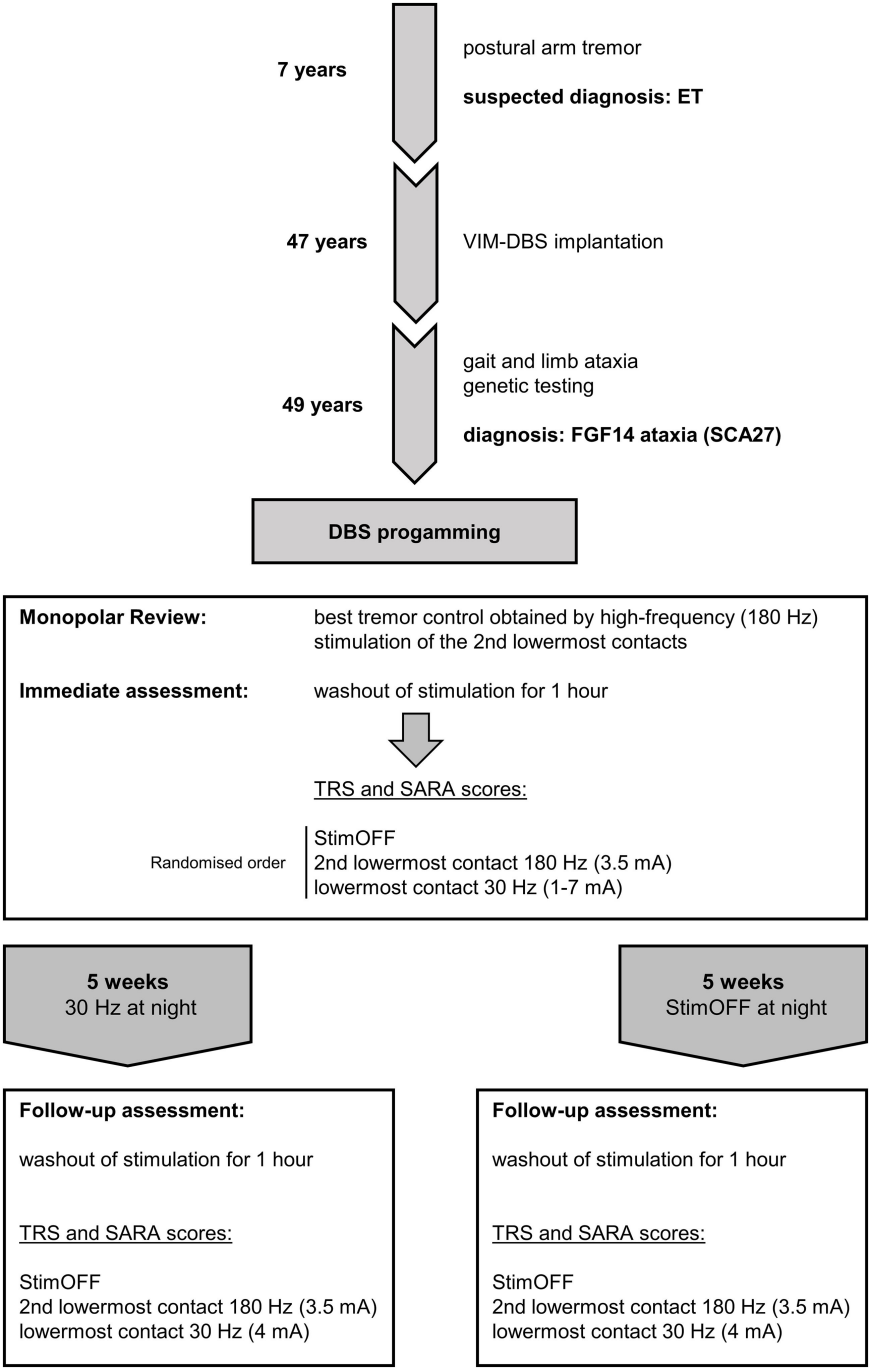
Timeline of the patient's history, diagnostic and therapeutic work-up. The patient was initially diagnosed with essential tremor (ET) due to early-onset postural arm tremor at the age of 7 years and implanted with VIM-DBS at the age of 47 years. Slowly emerging ataxia led to the diagnosis of spinocerebellar ataxia 27 (SCA 27) at the age of 49 years. Programming of deep brain stimulation (DBS) following a monopolar review showed relevant tremor attenuation by high-frequency stimulation of the 2nd lowermost contacts. The patient was randomized to follow-up stimulation settings meaning high-frequency stimulation at daytime and either StimOFF or 30 Hz stimulation of subthalamic fiber tracts (lowermost contacts) at night. Both stimulation paradigms were activated for 5 weeks in a randomized order followed by clinical examinations blinded to the settings of the previous interval.

Figure 2 and the according figure legend 2.

## Clinical assessment and DBS programming

**Figure 3:**
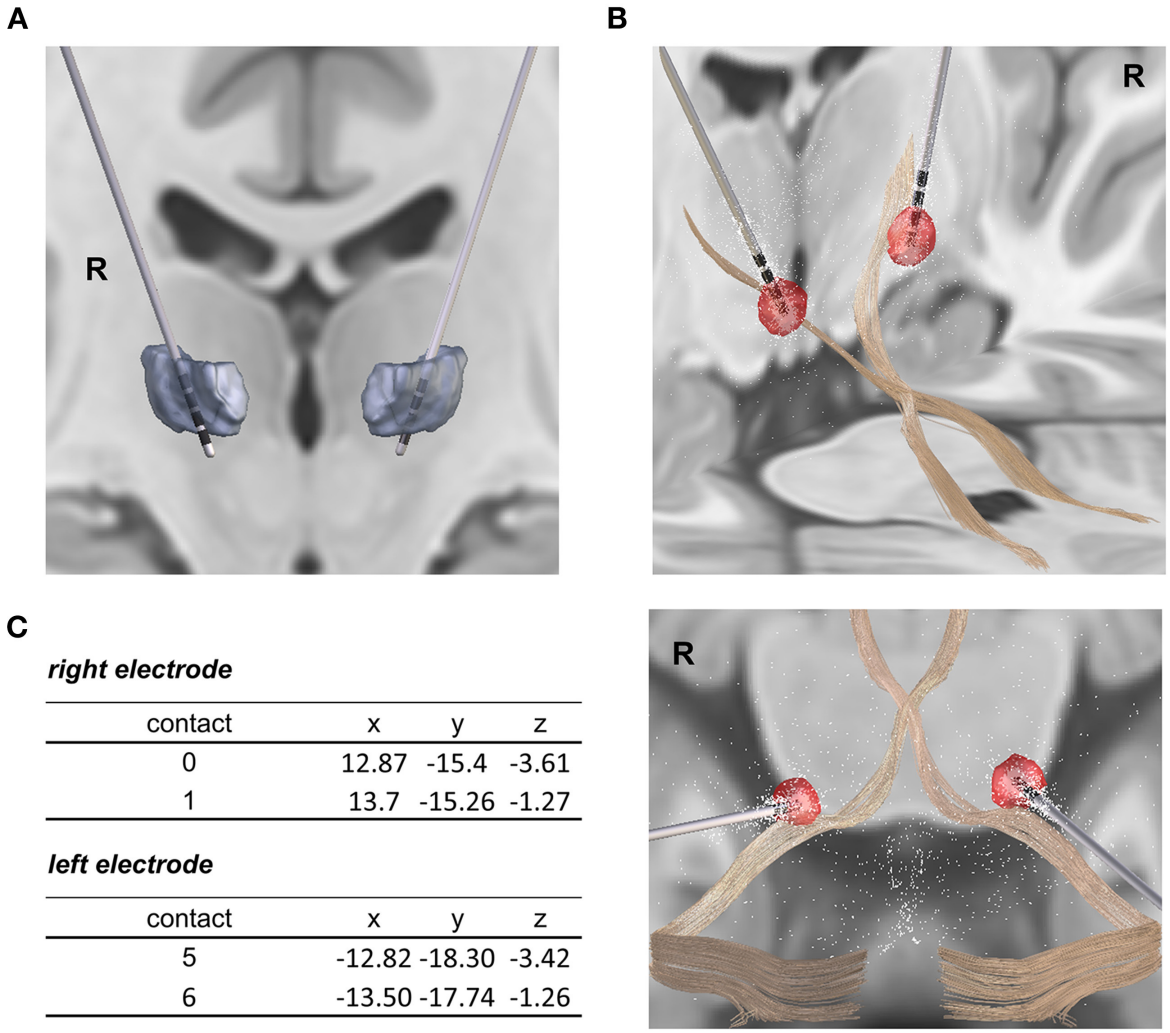
Reconstruction of DBS electrode placement. **(A)** Electrode placement in the VIM with the lowest contacts (0, 5) reaching beyond the thalamus border. **(B)** Topographic vicinity of the dentato-thalamic tract (DTT) and the volume of tissue activated (VTA) during low-frequency stimulation of the lowest contacts (30 Hz, 4 mA). The electrode position in relation to the DTT is shown from both a lateral and a superior perspective. The right side of the patient is marked with “R”. **(C)** Electrode placement and MNI coordinates of the active contacts for the high-frequency stimulation programme (contacts 1, 6: 3.5 mA, 30 Hz, 60 μs) and the low-frequency stimulation programme (contacts 0, 5: 4 mA, 30 Hz, 60 μs) are displayed. Electrode placement was reconstructed by co-registration of preoperative MRI and postoperative CT images and normalization into the MNI_ICBM_2009b_NLIN_ASYM space and anatomic atlases using the Lead-DBS toolbox.

**Figure 4:**
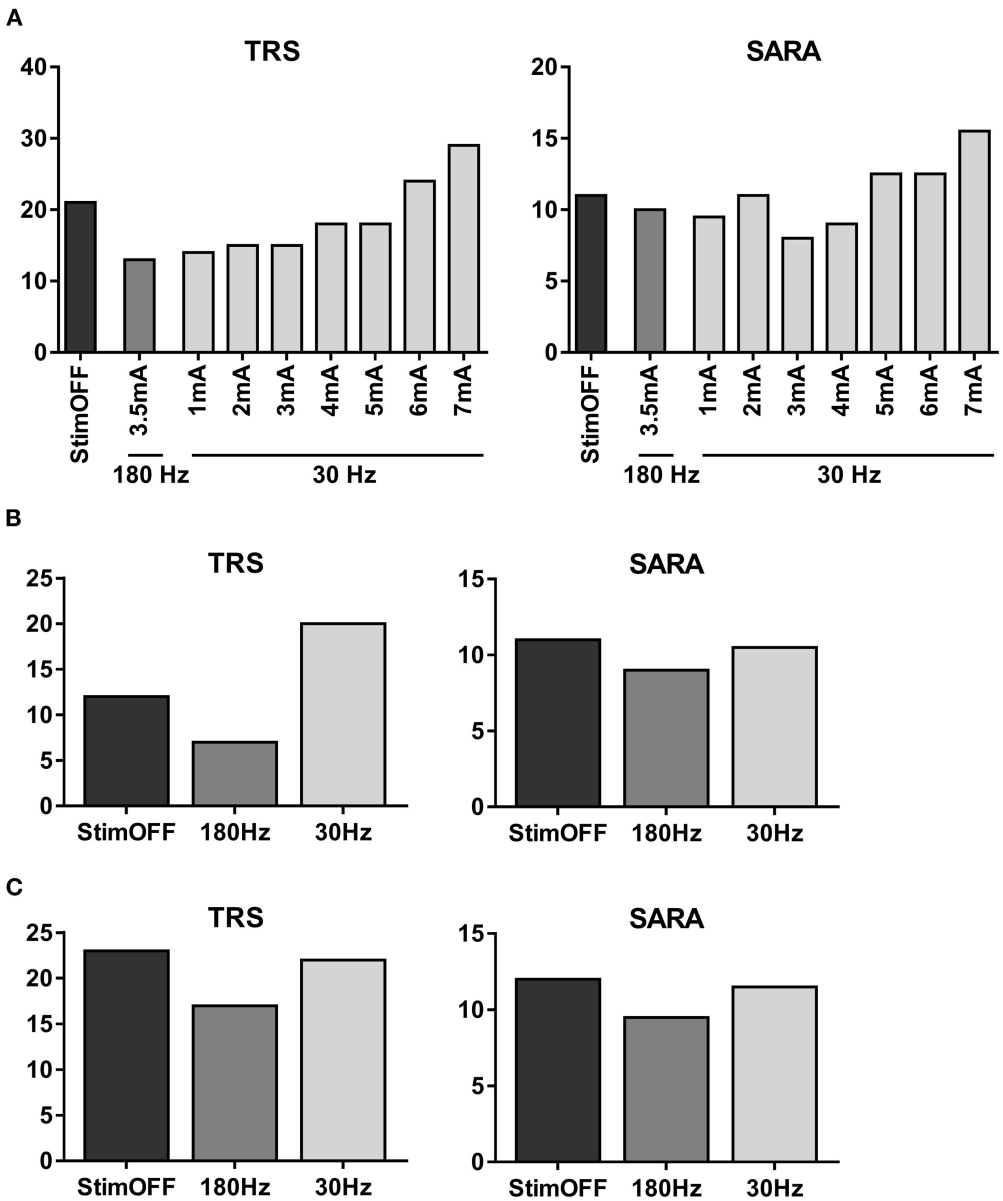
Clinical tremor and ataxia scores assessed with different stimulation paradigms. **(A)** In the immediate assessment, TRS and SARA were scored with StimOFF, 180 Hz-stimulation of the 2nd-lowermost (VIM) contacts and 30 Hz stimulation of the lowermost (subthalamic) contacts with increasing amplitudes from 1 to 7 mA in a randomized order. **(B)** The first follow-up examination was scheduled 5 weeks from baseline with preceding 180 Hz-stimulation (3.5 mA) at daytime and 30 Hz stimulation (4 mA) at night. **(C)** The second follow-up was scheduled 10 weeks from baseline with preceding 180 Hz-stimulation (3.5 mA) at daytime and StimOFF at night. For all assessments, the rater and patient were blinded to the respective previous stimulation settings.

As experimental models suggested benefits of low-frequency DN stimulation on ataxia and tremor (5), we performed differentiated assessments of subthalamic stimulation aiming for antidromic cerebellar neuromodulation. Before each programming session, DBS was turned off for 1 h in order to exclude a confounding rebound of tremor severity due to cessation of stimulation (3). In the immediate assessment, we reconfirmed that best tremor control was achieved by stimulation of the 2nd-lowermost contacts and stepwise ramping of the frequency up to 180 Hz increased this effect. Symmetrical stimulation settings were programmed in both hemispheres. Reconstruction of electrode placement indicated spatial vicinity between the DTT and the lowermost contacts (Figure 3). Therefore, the lowermost contacts were chosen for investigating the clinical effects of 30 Hz-stimulation with amplitudes ranging from 1 to 7 mA in a randomized order (Figure 4A). The highest amplitude tolerable for the patient (4 mA) was chosen for the follow-up. Simulation of the volume of tissue activated (VTA) by the 30 Hz-stimulation programme revealed a partial overlap of the VTA and the DTT (Figure 3B). In two follow-up intervals of 5 weeks, we used 180 Hz-stimulation of the 2nd-lowermost contacts for best tremor control at daytime. At night, 180 Hz-stimulation was turned off and the patient was instructed to use either StimOFF or 30 Hz-stimulation of the lowermost contacts. Investigator and patient were blinded to the night setting of the previous interval. Electrode placement and reconstruction of the VTA was conducted using the Lead-DBS toolbox (8) for Matlab (The MathWorks Inc., Natick, MA, USA) and anatomic atlases (9, 10). The Fahn-Tolosa-Marin-Tremor-Rating-Scale (TRS) items 1–9 and the Scale for the Assessment and Rating of Ataxia (SARA) were performed. Data analyses were conducted using GraphPad Prism 6.0 (GraphPad Software Inc, San Diego, CA, USA).

## Outcome

In the immediate assessment, tremor severity was attenuated by 180 Hz-VIM-stimulation compared to StimOFF (TRS 13 vs. 21). Tremor was also attenuated by 30 Hz-stimulation of the lowermost contact at 1 mA (TRS: 14), but gradually aggravated with stimulation amplitudes >4 mA (TRS up to 27). Changes of the SARA where mainly driven by the tremor response (Figure 4A).

During the first follow-up interval, the patient adopted intermittent 30 Hz-stimulation at night. Tremor in the StimOFF condition (TRS: 12) improved to a TRS of 7 by activating the 180 Hz-programme (Figure 4B). After the second interval during which the patient adopted StimOFF at night, the baseline TRS was higher in the StimOFF condition (TRS: 23) and slightly improved (TRS: 17) when using 180 Hz-stimulation (Figure 4C).

Thus, 180 Hz-stimulation at daytime and 30 Hz-stimulation at night led to a superior tremor response without occurrence of stimulation-induced aggravation of ataxia. In contrast, 180 Hz stimulation at daytime and StimOFF at night showed a decrease in tremor response.

## Discussion

Here we report the first case of a SCA 27 patient with favorable tremor response to DBS of the VIM and the subthalamic fiber tracts harboring the DTT. To date, a tremor-suppressing effect of high-frequency VIM-DBS was described in SCA 2, 3, 6, 31, and FXTAS (11–25) (Table 1). Stimulation of subthalamic projections provides the possibility of cerebellar neuromodulation by antidromic stimulation of the DTT (4, 26). We hypothesized that low-frequency stimulation of subthalamic projections may improve tremor and ataxia by entraining the cerebollo-thalamo-cortical network as suggested by experimental models in direct DN stimulation (5). Whilst night-time low-frequency stimulation of the lowermost contact, placed in the subthalamic area with close vicinity to the subthalamic fiber tracts including the DTT did not affect the ataxia outcome, a superior tremor response was observed. Therefore, treatment of tremor at daytime with high-frequency stimulation and prevention of habituation (3) and stimulation-induced ataxia (4) by intermittent low-frequency DBS may represent a novel approach.

However, this conclusion must take into account some limitations and pending issues, mainly based on the observation of a single patient in this case report. TRS scores in the StimOFF and StimON conditions after adopting intermittent low-frequency stimulation were considerably lower than after adopting StimOFF at night. Whether this difference may be attributed to a prolonged effect of the low-frequency stimulation cannot be conclusively determined on the basis of the observation of a single patient. Both conditions were tested at the same time of the day and in the same setting after a washout of stimulation for 1 h in order to minimize tolerance and rebound phenomena. Nevertheless, it is established that tremor severity is a fluctuating symptom affected by various confounders like anxiety and the overall noradrenergic tone which were not controlled in this case study. Moreover, entrainment of cerebello-thalamo-cortical networks by neuromodulation of the DTT was only assumed by a normative connectomic approach as recently adopted in other studies (7). Simulation of the VTA of the 30 Hz low frequency programme partially covered fibers of the DTT, but it should be considered that the stimulation programme tested may not have affected the entire DTT. The cerebello-thalamo-cortical networks involved are located within the “anatomical bottle-neck” (6) of the subthalamic area and the adjacent thalamus. Both lower electrode contacts tested here potentially interfere with these networks. Eventually, further electrophysiological or functional imaging data are required to assess the neurophysiological mechanisms behind the effects reported in this case report.

Here we report the first case of a SCA 27 patient experiencing relevant symptom relieve by DBS of the VIM and subthalamic fiber tracts including the DTT. Moreover, as before only described in experimental models the approach of low-frequency stimulation of the cerebello-thalamo-cortical network resulted in a superior tremor response. On this basis, stringent clinical studies may tie in and provide a new therapeutical perspective for patients with tremor and ataxia in SCA 27 and beyond.

## Data availability statement

The raw data supporting the conclusions of this article will be made available by the authors, without undue reservation.

## Ethics statement

Ethical review and approval was not required for the study on human participants in accordance with the local legislation and institutional requirements. The patients/participants provided their written informed consent to participate in this study. Written informed consent was obtained from the individual(s) for the publication of any potentially identifiable images or data included in this article.

## Author contributions

ML: research project: conception, organization, execution, and manuscript preparation: writing of the first draft. MS: research project: conception, and manuscript preparation: review and critique. IC, PK, MH, TG, and AG: research project: organization, and manuscript preparation: review and critique. DW: research project: conception, organization, execution, and manuscript preparation: review and critique. All authors contributed to the article and approved the submitted version.

## References

[1] Groth CL Berman BD. Spinocerebellar ataxia 27: a review and characterization of an evolving phenotype. Tremor Other Hyperkinet Mov. (2018) 8:325. 10.5334/tohm.436 29416937 PMC5801325

[2] Kvernmo N Konglund AE Reich MM Roothans J Pripp AH Dietrichs E. Deep brain stimulation for arm tremor: a randomized trial comparing two targets. Ann Neurol. (2022) 91:585–601. 10.1002/ana.26317 35148020 PMC9311445

[3] Paschen S Forstenpointner J Becktepe J Heinzel S Hellriegel H Witt K. Long-term efficacy of deep brain stimulation for essential tremor: an observer-blinded study. Neurology. (2019) 92:e1378–86. 10.1212/WNL.0000000000007134 30787161

[4] Reich MM Brumberg J Pozzi NG Marotta G Roothans J Åström M. Progressive gait ataxia following deep brain stimulation for essential tremor: adverse effect or lack of efficacy? Brain. (2016) 139:2948–56. 10.1093/brain/aww223 27658421

[5] Anderson CJ Figueroa KP Dorval AD Pulst SM. Deep cerebellar stimulation reduces ataxic motor symptoms in the shaker rat. Ann Neurol. (2019) 85:681–90. 10.1002/ana.25464 30854718 PMC8098166

[6] Fox MD Deuschl G. Converging on a neuromodulation target for tremor. Ann Neurol. (2022) 91:581–4. 10.1002/ana.26361 35362142

[7] Nowacki A Barlatey S Al-Fatly B Dembek T Bot M Green AL. Probabilistic mapping reveals optimal stimulation site in essential tremor. Ann Neurol. (2022) 91:602–12. 10.1002/ana.26324 35150172

[8] Horn A Kühn AA. Lead-DBS: a toolbox for deep brain stimulation electrode localizations and visualizations. Neuroimage. (2015) 107:127–35. 10.1016/j.neuroimage.2014.12.002 25498389

[9] Middlebrooks EH Domingo RA Vivas-Buitrago T Okromelidze L Tsuboi T Wong JK. Neuroimaging advances in deep brain stimulation: review of indications, anatomy, and brain connectomics. AJNR Am J Neuroradiol. (2020) 41:1558–68. 10.3174/ajnr.A6693 32816768 PMC7583111

[10] Ewert S Plettig P Li N Chakravarty MM Collins DL Herrington TM. Toward defining deep brain stimulation targets in MNI space: a subcortical atlas based on multimodal MRI, histology and structural connectivity. Neuroimage. (2018) 170:271–82. 10.1016/j.neuroimage.2017.05.015 28536045

[11] Freund H-J Barnikol UB Nolte D Treuer H Auburger G Tass PA. Subthalamic-thalamic DBS in a case with spinocerebellar ataxia type 2 and severe tremor: a unusual clinical benefit. Mov Disord. (2007) 22:732–5. 10.1002/mds.21338 17265523

[12] Oyama G Umemura A Shimo Y Nishikawa N Nakajima A Jo T. Posterior subthalamic area deep brain stimulation for fragile X-associated tremor/ataxia syndrome. Neuromodulation. (2014) 17:721–3. 10.1111/ner.12150 24528808

[13] Isobe T Sato H Goto T Yako T Yoshida K Hashimoto T. Long-term suppression of disabling tremor by thalamic stimulation in a patient with spinocerebellar ataxia type 2. Stereotact Funct Neurosurg. (2019) 97:241–3. 10.1159/000504062 31743916

[14] Cury RG França C Duarte KP Paraguay I Diniz JM Cunha P. Safety and outcomes of dentate nucleus deep brain stimulation for cerebellar ataxia. Cerebellum. (2021) 21:861–5. 10.1007/s12311-021-01326-8 34480330

[15] Cury RG França C Silva V Barbosa ER Capato TTC Lepski G. Effects of dentate nucleus stimulation in spinocerebellar ataxia type 3. Parkinsonism Relat Disord. (2019) 69:91–3. 10.1016/j.parkreldis.2019.10.029 31706132

[16] Kuo MC Tai CH Tseng SH Wu RM. Long-term efficacy of bilateral subthalamic deep brain stimulation in the Parkinsonism of SCA 3: a rare case report. Eur J Neurol. (2022) 29:2544–7. 10.1111/ene.15339 35837753

[17] Hashimoto T Muralidharan A Yoshida K Goto T Yako T Baker KB. Neuronal activity and outcomes from thalamic surgery for spinocerebellar ataxia. Ann Clin Transl Neurol. (2018) 5:52–63. 10.1002/acn3.508 29376092 PMC5771317

[18] Hamel W Herzog J Kopper F Pinsker M Weinert D Müller D. Deep brain stimulation in the subthalamic area is more effective than nucleus ventralis intermedius stimulation for bilateral intention tremor. Acta Neurochir. (2007) 149:749–58; discussion 758. 10.1007/s00701-007-1230-1 17660940

[19] Weiss D Mielke C Wächter T Bender B Liscic RM Scholten M. Long-term outcome of deep brain stimulation in fragile X-associated tremor/ataxia syndrome. Parkinsonism Relat Disord. (2015) 21:310–3. 10.1016/j.parkreldis.2014.12.015 25577024

[20] Ferrara JM Adam OR Ondo WG. Treatment of fragile-X-associated tremor/ataxia syndrome with deep brain stimulation. Mov Disord. (2009) 24:149–51. 10.1002/mds.22354 18951504

[21] Senova S Jarraya B Iwamuro H Tani N Ouerchefani N Lepetit H. Unilateral thalamic stimulation safely improved fragile X-associated tremor ataxia: a case report. Mov Disord. (2012) 27:797–9. 10.1002/mds.24923 22287158

[22] Xie T Goodman R Browner N Haberfeld E Winfield L Goldman J. Treatment of fragile X-associated tremor/ataxia syndrome with unilateral deep brain stimulation. Mov Disord. (2012) 27:799–800. 10.1002/mds.24958 22344717

[23] Mehanna R Itin I. Which approach is better: bilateral vs. unilateral thalamic deep brain stimulation in patients with fragile X-associated tremor ataxia syndrome. Cerebellum. (2014) 13:222–5. 10.1007/s12311-013-0530-7 24122741

[24] dos Santos Ghilardi MG Cury RG dos Ângelos JS Barbosa DC Barbosa ER Teixeira MJ. Long-term improvement of tremor and ataxia after bilateral DBS of VoP/zona incerta in FXTAS. Neurology. (2015) 84:1904–6. 10.1212/WNL.0000000000001553 25862802

[25] Tamás G Kovács N Varga NÁ Barsi P Eross L Molnár MJ. Deep brain stimulation or thalamotomy in fragile X-associated tremor/ataxia syndrome? Case report. Neurol Neurochir Pol. (2016) 50:303–8. 10.1016/j.pjnns.2016.04.004 27375149

[26] Groppa S Herzog J Falk D Riedel C Deuschl G Volkmann J. Physiological and anatomical decomposition of subthalamic neurostimulation effects in essential tremor. Brain. (2014) 137(Pt 1):109–21. 10.1093/brain/awt304 24277721

